# Genome-resolved metagenomic survey of 500 samples from 56 hot springs across the Western US

**DOI:** 10.1038/s41597-026-07139-w

**Published:** 2026-04-01

**Authors:** Masha V. Korchagina, Claire E. Mullin, Hengameh H. Soufi, Sophia Lambert, Ines G. Moran, Robert Porch, Sage E. Albright, Alexandria S. Doran, Leila M. Jones, Nathan Malamud, Qusheng Jin, A. Michelle Wood, Stilianos Louca

**Affiliations:** 1 Department of Biology, University of Oregon, Eugene, USA; 2 Institute of Ecology and Evolution, University of Oregon, Eugene, USA; 3 Department of Plant and Soil Sciences, University of Kentucky, Lexington, USA; 4 Wildlife Bioacoustics Analytics, Vancouver, Canada; 5 Department of Earth Sciences, University of Oregon, Eugene, USA

## Abstract

Hot springs are natural laboratories for studying microbial diversity, evolution, and adaptation to extreme environments. Despite their abundance across the Western US, information about the functional and genomic structure of inhabiting microbial communities is restricted to a handful of locations. Here we present a dataset of 500 deep metagenomes, totaling 3.38 terabasepairs and collected from 56 remote hot springs across the US Great Basin and Yellowstone, with 25 of the hot springs surveyed annually over 4 consecutive years. Additionally, we present 780 bacterial and archaeal metagenome-assembled genomes (MAGs) binned from these metagenomes, with completeness ≥80% and contamination ≤5%, of which 149 are considered “high quality”. Many of the MAGs likely represent entirely novel genera and even families, relative to the Genome Taxonomy Database. Our spatiotemporally extensive dataset yields insight into the microbial functional structure at dozens of previously unstudied locations, substantially expands our repertoire of extremophile microbial genomes, provides a new resource for high-temperature biotechnology, and enables future phylogenomic studies of these communities through space and time.

## Background & Summary

Hot springs are extreme environments that provide a unique window into the ecology and evolution of microbial life, and hold great promise for discovering novel and industrially significant microbial taxa. Indeed, these environments often exhibit high temperatures and extreme pH, thus exerting strong selective pressures on microorganisms that lead to unique adaptations. For example, the industrially important heat-stable *Taq* polymerase was first isolated from a hot spring bacterium^1^. Due to the contrast between hot springs and their surrounding environments, they resemble “isolated islands” from the perspective of resident microbes, which exhibit particularly strong geographic isolation^2,3^ and may even speciate within individual hot springs^4^. Hot springs and other geothermal environments also likely resemble some of the earliest life-harboring environments on Earth^5,6^, thus yielding insights into the early evolution of life.

In large stretches of the Western United States—particularly the Great Basin and Yellowstone — tectonic deformation, magmatic activity and crustal heat flow have given rise to thousands of hot springs, making these regions among the most geothermally active in the world.^7,8^. Yet, with the exception of Yellowstone, few studies exist on the microbial diversity in this region, and existing studies are mostly restricted to morphological descriptions and 16S rRNA amplicon sequences^9–12^. Hot springs in the Great Basin are expected to be especially isolated—from one another and from geothermal systems in other regions—due to the arid, desert-like surrounding environment, which poses a strong dispersal barrier for hot spring-adapted microorganisms. This geographic and ecological isolation likely contributes to their distinct and often highly endemic microbial diversity. As a case in point, a previous survey of bacterial and archaeal (henceforth “prokaryotic”) 16S rRNA sequences in Little Hot Creek hot springs (California Great Basin)^11^ found that over half of the prokaryotic phylotypes could not be grouped into known taxonomic classes. Similarly, a survey of hot springs in the northwestern Great Basin found that more than one third of bacterial 16S rRNA sequences could not be matched to any cultivated phyla^10^. Incidentally, the rapid spread of geothermal power plants in the Western US^7,13,14^ causes a “drying” of an increasing number of hot springs, potentially leading to an irreversible loss of endemic microbial diversity that could forever remain undiscovered.

**Fig. 1:**
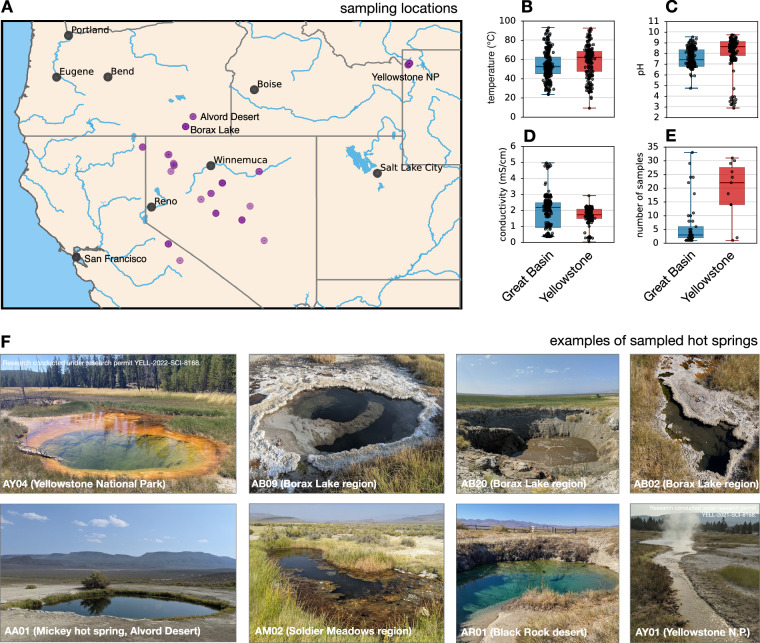
Overview of samples. (**A**) Geographic locations of the 56 surveyed hot springs (purple circles). Note that many hot springs overlap on the map due to their proximity, and that in most cases multiple samples were collected from each hot spring. (**B–D**) Physicochemical variables measured in-situ at each sampling location at the time of sampling (one point per sample). (**E**) Number of samples collected per hot spring (one point per hot spring). (**F**) Photos of some representative hot springs surveyed. Photos by S.L.

Modern metagenomic sequencing, especially the ability to generate metagenome-assembled genomes (MAGs), is revolutionizing environmental microbiology, yielding insight not just into the taxonomic identities but the metabolic capabilities and ecological strategies of populations^15^. Here we present a dataset of 500 deeply sequenced (shotgun) metagenomes generated from biological samples, collected from 56 hot springs and their proximate outflows across the Great Basin and Yellowstone over the course of 4 years (Fig. 1). All surveyed hot springs are relatively pristine with little to no human infrastructure, and are located in remote or sparsely populated areas such as Black Rock desert, Soldier Meadows and Dixie Valley in Nevada, Alvord desert and Borax Lake region in Oregon, Upper Alkali Lake and the eastern flanks of the Sierra Nevada in California, and Yellowstone National Park in Wyoming. 25 of the hot springs were surveyed annually on 4 consecutive years, while 29 were surveyed only once. An average of 9 distinct samples were collected per hot spring to account for local heterogeneities in physicochemical conditions and microbial compositions, with some larger hot springs represented by over 30 samples (Fig. 1E). We also present 780 bacterial and archaeal metagenome-assembled genomes recovered from these samples, spanning over 16 phyla and exhibiting a wide array of metabolic capabilities, ranging from oxygenic and anoxygenic phototrophy, fermentation and reductive acetogenesis to respiration of nitrogen and sulfur compounds. In addition to the microbiome surveys, we also measured in-situ water temperature, pH and electrical conductivity (Fig. 1B–D and File “Data_01 - Sample_metadata.tsv” deposited on Figshare^16^), all three of which are known to be among the strongest factors influencing microbial community composition in hot springs^8,17^ and other aquatic environments^18,19^. These measurements were performed at each sampling location at the time of sampling, i.e., representing nearly 3 × 500 measurements (with the exception of a small number of technical failures).

## Methods

### Sample collection and measurements

Springs were chosen based on multiple criteria, preferably having source temperatures above the 36.7°C threshold proposed for hot springs by^20^ (with some exceptions), remoteness and pristinity, none or minimal infrastructure on the spring itself, accessibility by vehicle within 1 km, previous proof of existence (such as chemical measurements by USGS, or sightings by the public), sufficient size to ensure persistence at least over the course of this study, broad geographic coverage across the Western US and ease of obtaining sampling permits. In total 56 springs were chosen for surveying (map in Fig. 1A,F), covering the states of California, Oregon, Nevada and Wyoming. Nearly all springs (53 out of 56) had source temperatures above the 36.7°C threshold in at least one survey. The remaining 3 springs were included due to their proximity to other much hotter springs, likely sharing a common underground water source and being of potential ecological/evolutionary importance in the region. These included springs AB20 and AB35 near Borax Lake, Oregon (maximum source temperatures 27.5°C and 36.5°C, respectively), as well as spring AW03 in Buffalo Valley, Navada (28.8°C). For simplicity, all springs are henceforth referred to as hot springs. We stress that, since hot springs were inevitably selected based on a combination of physical, geographical and practical criteria, including size, accessibility and permitting constraints, our dataset does not constitute a completely random and unbiased sample of hot springs or geothermal biodiversity across the Western US; this limitation should be kept in mind in future analyses.

Surveys were performed over the course of 4 years during the months of June to September for logistical reasons, with any given hot spring visited at most once each year. Due to variations in funding and personnel availability, the number of hot springs surveyed differed between years, with priority generally placed on larger and more easily accessible hot springs with a lower likelihood of being dry while also maximizing consistency between years. Some hot springs were opportunistically included once for additional research directions. Hence, 25 hot springs were surveyed annually over all 4 years, 2 hot springs were surveyed 3 times, 1 hot spring was surveyed twice, and 29 hot springs were surveyed only once. Detailed survey dates are listed in “Data_01 - Sample_metadata.tsv”. Permission to sample was ensured for all sites (see Acknowledgments).

Depending on hot spring size and visually inferred local microbial diversity, one or more locations were sampled at each hot spring during any given survey. Locations within a hot spring were chosen broadly across the hot spring to maximize the diversity covered (Fig. 1E), but independently each year depending on local conditions. Note that many hot springs generated a runoff stream and that samples were also collected from these streams in the vicinity of the hot springs (no more than 100 m). Some sampling locations thus exhibited substantially lower temperatures than the corresponding hot spring source. At each sampling location, we first measured water temperature, pH and electrical conductivity using an Oakton PC 450 multimeter (Fig. 1B–D). Note that for a small number of samples some parameters could not be measured due to the shallowness or turbidity of the water. We then collected up to 2 mL of microbial mat, filament, sediment or mud material at each site for biological analysis, using a spatula attached to an extension pole where necessary. Material was always collected from the top 1–10 cm depending on substrate thickness, softness and structure (e.g., filaments versus hard crust), stored in sterile cryotubes and flash-frozen in a dry shipper at liquid-nitrogen temperature until further processing. Geographic coordinates of individual samples were recorded using the GPS function of a smartphone, and thus may exhibit an uncertainty of a few meters. We emphasize that the present study focuses on molecular sequence data and thus only includes a limited number of environmental measurements. That said, users that wish to examine biological/biogeographic patterns in relation to more detailed environmental conditions could augment our measurements with macro-scale climatic and geological data from public databases such as the NASA Earth Observations gridded database^21^ or the Global Lithological Map database^22^.

### DNA extraction and sequencing

DNA was extracted from each of the 500 samples using the DNeasy^™^ PowerSoil Pro or DNeasy^™^ PowerBiofilm kit, depending on the material, closely following the manufacturer’s standard protocol. Shotgun metagenomic sequencing of the extracted DNA was either performed by the Integrated Microbiome Resource in Dalhousie (IMR, years 2020–2021) or the University of Minnesota Genomics Center (UMGC, years 2022–2023). At IMR, metagenomic libraries were prepared using the Illumina Nextera Flex kit and sequenced using an Illumina NextSeq 2000 (2 × 150 bp paired ends). At UMGC, metagenomic libraries were prepared using the Illumina Nextera XT kit and sequenced using an Illumina NovaSeq X Plus (2 × 150 bp paired reads). A total of 11,485,591,914 sequence pairs or  ~ 3.38 Tbp of data were generated (Fig. 3A).

### Metagenomic analyses

**Fig. 2:**
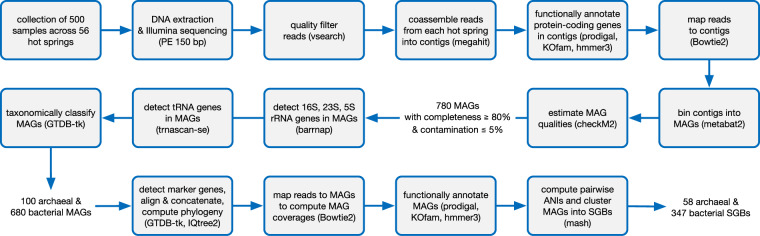
Overview of workflow. Overview of the main bioinformatics workflow followed in this study, including the recovery of metagenome-assembled genomes (MAGs) and their clustering into species-level genome bins (SGBs).

**Fig. 3:**
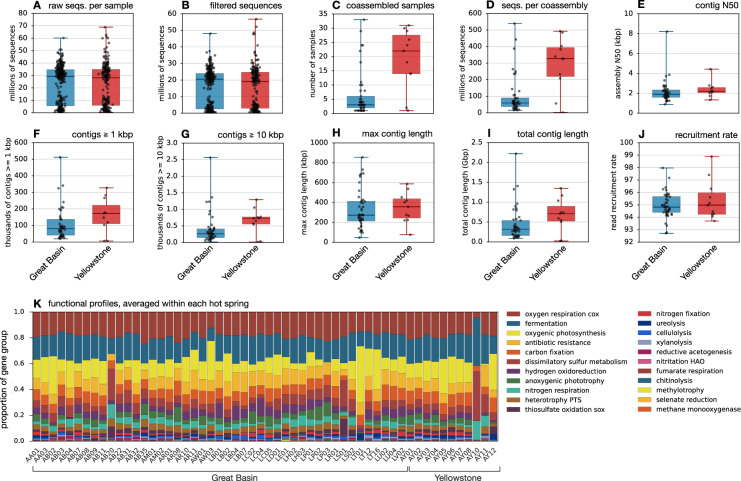
Overview of metagenomes. (**A–J**) Box plots of (**A**) the numbers raw metagenomic sequences per sample, (**B**) the number of quality-filtered metagenomic sequences per sample, (**C**) the number of samples per coassembly, (**D**) the number of input sequences per coassembly, (**E**) the contig N50 per coassembly, (**F**) the number of contigs with lengths ≥1 kbp per coassembly, (**G**) the number of contigs with lengths ≥10 kbp per coassembly, (**H**) the maximum contig length per coassembly, (**I**) the total contig length per coassembly and (**J**) the read recruitment rate (fraction of properly paired reads mapped) per coassembly. All box plots are split by broad geographic region. (**K**) Estimated proportions of various gene groups of ecological interest, averaged within each hot spring and estimated based on reads mapped.

An overview of our bioinformatics workflow is given in Fig. 2. Unless mentioned otherwise, software tools were used with default parameters. Adapters were trimmed from reads using the tool skewer v0.2.2^23^ with option “–mode pe”. Reads were then quality-filtered using vsearch v2.28.1^24^ with options “–fastq_ascii 33 –fastq_maxee 0.5 –fastq_truncee 0.5 –fastq_qmax 64 –fastq_maxee_rate 0.002 –fastq_stripleft 0 –fastq_trunclen_keep 10000”, retaining 7,778,111,627 high-quality read pairs or  ~ 1.799 Tbp (Fig. 3B). Our quality filtering was intentionally aggressive, as we prioritized a high quality of retained reads and a lower subsequent error rate in constructed contigs. For each hot spring, sequences from all associated samples were pooled and coassembled into longer contiguous sequences (contigs) using megahit v1.2.9^25^ with option “–min-contig-len 500”. A total of 17,918,724 contigs were generated, with an average length of 1503 bp and a maximum length of 854,726 bp (overview in Fig. 3C–I). The read recruitment rate (Fig. 3J) was computed for each hot spring by mapping the quality-filtered reads from that hot spring to the corresponding coassembled contigs using Bowtie2 v2.5.1^26^ with option “–no-unal”, and then extracting the “properly paired” summary statistic with samtools flagstat v1.17^27^. We mention that alternative (co)assembly strategies may be envisioned depending on research objectives, for example by geographic region or separately for each sample, each with certain trade-offs^28–30^; we thus provide our full raw sequence data so that users may explore alternative strategies if needed. An overview of summary statistics related to the metagenomic sequences is given in Fig. 3A–J.

Gene-centric functional profiles (Fig. 3K) were generated as described previously^31,32^. Here we thus only provide a brief summary. For any given contig, contig proportions were computed by mapping the sample’s non-assembled quality-filtered reads to the contigs using Bowtie2, then counting the number of reads mapped to each contig with a MAPQ score ≥30, subsequently dividing that number by the contig length, and finally normalizing the obtained ratios in each sample to sum 1. Protein-coding genes (PCGs) were predicted in the contigs using prodigal v2.6.3 with option “-p meta”^33^. PCGs were then mapped to KEGG gene orthologs (KOs) in the KOfam HMM database r111^34^ using hmmsearch v3.3^35^. Only hits with an E-value below 10^−10^ were considered. For each sample, the proportions of PCGs were computed by first associating with each PCG the proportion of its host contig, and then normalizing those values in each sample to sum 1. The proportion of a given KO in a given sample was estimated by summing the proportions of all PCGs mapped to that KO. Lastly, KOs were aggregated into higher-level functional groups of ecological interest as described previously^31,32^.

### Metagenome-assembled genomes

**Fig. 4:**
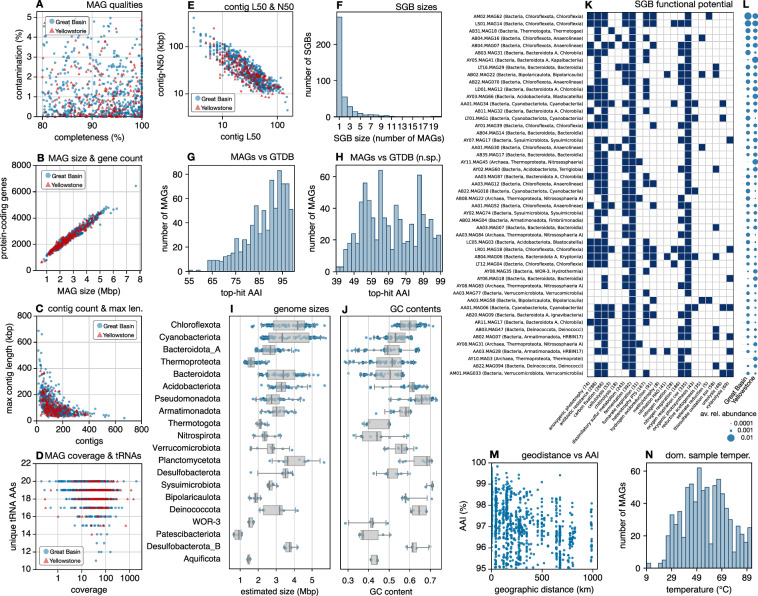
Overview of metagenome-assembled genomes. (**A**) Completeness and contamination levels of MAGs, one point per MAG, styled by broad geographic region (Great Basin vs Yellowstone). (**B**) MAG sizes (total contig length) and numbers of detected protein-coding genes. (**C**) Number of contigs and maximum contig length per MAG. (**D**) MAG coverages (number of read basepairs mapped to each MAG basepair, considering only reads from a MAG’s origin hot spring) and number of unique amino-acids for which elongator tRNAs were detected, per MAG. (**E**) Contig L50 and N50 per MAG. (**F**) Distribution of SGB sizes. (**G**) Top average amino-acid identity (AAI) between each MAG and any GTDB reference genome. (**H**) Similar to L, but restricted to reference genomes with a full species name. (**I**) Box-plot of estimated genome sizes of populations represented by MAGs (one point per MAG), correcting for MAG completeness, separated by phylum. (**J**) Similar to I, but showing GC contents of MAGs. (**K**) Functional potential of the 50 most relatively abundant SGBs, based on the presence (blue) or absence (white) of various gene groups of ecological importance (one row per SGB, one column per gene group). The total number of SGBs positive in any given gene group is indicated in parentheses. (**L**) Average relative abundance of each SGB shown in (**K**), i.e., averaged across samples, separately across Great Basin samples or Yellowstone samples. (**M**) Pairwise AAIs between closely related MAGs (AAI ≥95%) compared to pairwise geographic distances (one point per MAG pair). (**N**) Histogram of the dominant sample temperatures across MAGs, i.e., of the temperature measured at the sample in which each MAG had highest relative abundance.

MAGs were constructed from the coassembled contigs, separately for each hot spring, as follows. For each sample, reads were mapped back to contigs using Bowtie2 with option “–no-unal”. SAM files generated by Bowtie2 were converted to BAM files using samtools v1.17. BAM files and contig fasta files were used as input to MetaBAT2 v2.15-2^36^ for binning contigs into MAGs, with options “-m 5000 -s 200000”. This yielded a separate set of MAGs for each coassembly, i.e., for each hot spring. MAG completeness and contamination were estimated using CheckM2 v1.0.1^37^ (Fig. 4A). Henceforth, only MAGs with a completeness of at least 80% and a contamination of no more than 5% were kept (780 MAGs). An overview of retained MAGs is given in Fig. 4. MAGs were taxonomically classified with the GTDB-Tk toolkit, workflow classify_wf v2.3.0^38^ using GTDB r226. This yielded 680 bacterial and 100 archaeal MAGs. Alignments of multiple single-copy universal marker genes generated by GTDB-Tk were concatenated and used to separately construct a phylogeny of bacterial and archaeal MAGs with IQtree2 v2.4.0^39^ with automatic model selection. Support values for tree nodes were computed using the IQtree2 option “-B 1000”, which invokes the ultrafast bootstrap algorithm^40^. The archaeal tree was rooted such that *Euryarchaeota* MAGs (covering the *Halobacteriota* and *Thermoplasmatota*) become a basal clade^41^. The bacterial tree was rooted using the Minimum Variance method by^42^. For visualization purposes (Fig. 6), the archaeal and bacterial trees were connected at their respective roots. The combined tree was made ultrametric for visualization purposes based on relative evolutionary divergence using the function date_tree_red in the R package castor v1.8.4^43^. In each MAG, rRNA genes (16S, 23S and 5S) were detected using barrnap v0.9, and tRNA genes were detected using trnascan-se v2.0.12^44^. Protein-coding genes were detected in each MAG using prodigal^33^ with option “-p single”, and functionally annotated using hmmsearch^35^ and KOfam^34^ as described earlier for the coassemblies.

**Fig. 5:**
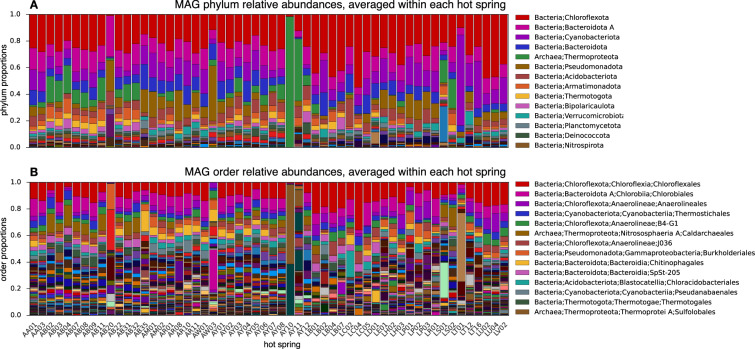
Relative taxon abundances. (**A**) Estimated average relative abundances of phyla in various hot springs, based on MAG relative abundances (averaged within each hot spring) and taxonomic classifications of MAGs. Each color represents a different phylum, and the size of a color bar is proportional to a phylum’s average relative abundance in all samples collected from a given hot spring. Only the most abundant taxa are listed in the legend to avoid clutter. Note that since recovered MAGs only represent a subset of a community, these taxonomic profiles only partially reflect the true taxonomic diversity. (**B**) Similar to A, but showing estimated average relative abundances of orders.

To estimate the relative abundances of MAGs in each sample, and to estimate their coverages by metagenomic reads, we proceeded as follows. We first competitively mapped the quality-filtered reads from each sample to the MAGs using Bowtie2, retaining only alignments with a MAPQ score ≥30. For any given MAG and sample, we then computed the coverage at each nucleotide position using samtools depth and then averaged those coverages across the entire MAG. For computational efficiency, only a random subset of at most 100,000 read-pairs from each sample were mapped, and coverages were corrected to account for these rarefactions. The relative abundance of a MAG in a given sample was computed by dividing its coverage in that sample by the sum of coverages of all MAGs in that sample. The total coverage of a MAG, i.e. the number of read basepairs from its origin hot spring mapped to each basepair (Fig. 4D), was computed by summing its sample-specific coverages over all samples in its origin hot spring. The relative abundances of higher-order taxa, such as phyla or orders, were estimated by summing the relative abundances of recovered MAGs within each taxon (Fig. 5). To estimate the extent to which our MAGs represent the original metagenomes, we calculated the fraction of quality-filtered reads from each sample that were mapped to any of the MAGs with a MAPQ score ≥30; on average 13% of reads could be mapped per sample, with the lowest rate being 0.8% and the highest rate being 78% depending on the sample.

**Fig. 6:**
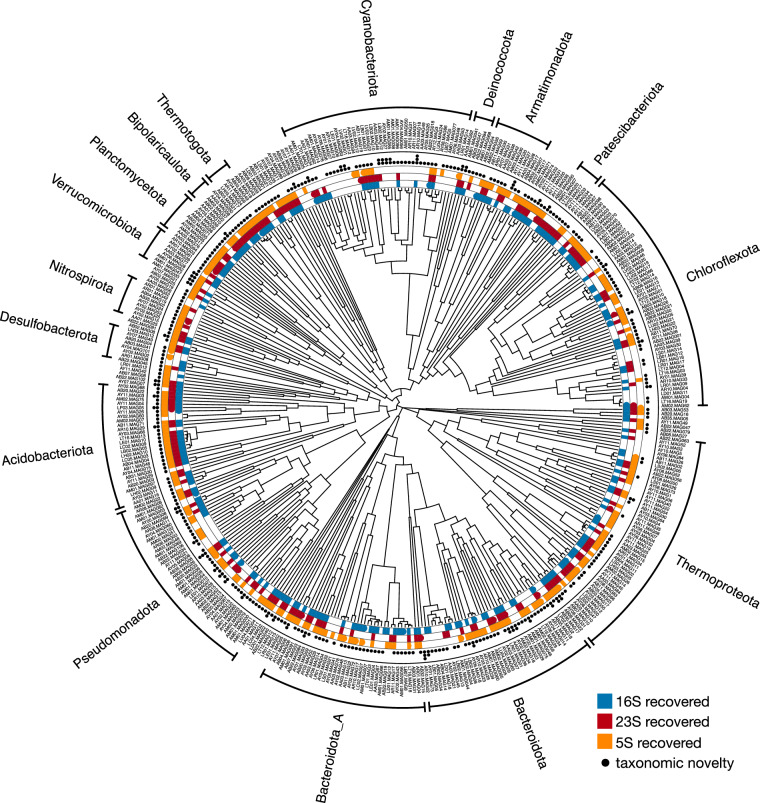
SGB phylogeny. Multi-gene phylogeny of SGB representatives, with presence/absence of 16S/23S/5S rRNA genes (color segments) and taxonomic novelty (dots) indicated along the perimeter. One, two or three dots indicate that the SGB could only be placed into a known genus, family or order, respectively, using a joint phylogeny with the Genome Taxonomy Database and allowing candidatus and other informal taxa. Major phyla (i.e., with at least 5 SGBs) are indicated in the perimeter. The phylogeny was made ultrametric for visualization purposes based on relative evolutionary divergence.

To determine the species-level diversity in our MAG set, we clustered MAGs into SGBs based on an average nucleotide identity (ANI) cutoff of 95%, following general convention^45,46^ and as described previously^3,47^. Briefly, pairwise ANIs between MAGs were computed using fastANI v1.33^45^, based on which MAGs were hierarchically clustered using fastcluster v1.3.0^48^. Tips in the hierarchical clustering trees were grouped into SGBs based on a minimum pairwise ANI of 95%, using the function collapse_tree_at_resolution in castor^43^, and a single representative MAG with highest estimated completeness was kept from each resulting cluster (details in^3,47^). An overview of SGB sizes is shown in Fig. 4F. The phylogenetic tree in Fig. 6 was visualized in R using the castor package^43^. All other visualizations were generated in python using the matplotlib package v3.8.3^49^.

To examine the taxonomic novelty of our MAGs, we constructed joint multi-gene phylogenetic trees of the MAGs with GTDB r226 reference genomes using the GTDB-Tk de_novo_wf workflow^38^, separately for archaea and bacteria. We used the phyla *Altiarchaeota*^50^ and *Patescibacteriota*^51,52^ as basal clades to root the archaeal and bacterial trees, respectively. We then recorded the lowest possible named taxon into which each MAG was placed by GTDB-Tk. For example, if the lowest-level taxon name among all ancestral nodes to a MAG was a class, then this was taken as an indication that the MAG belongs to a novel order relative to the GTDB. To further asses the novelty of MAGs relative to the GTDB r226, we also computed all pairwise AAIs between MAGs and GTDB reference genomes using mash v2.3^53^, and for each MAG kept a record of the closest hit, i.e., with the highest AAI (Fig. 4G). To specifically assess novelty relative to properly named species, we repeated this computation while restricting it to only GTDB reference genomes with a full species name (Fig. 4H). To visualize the relationship between MAG relatedness and their geographic distance, which may be indicative of dispersal barriers, we plotted pairwise AAIs between closely related MAGs against pairwise geographic distances (Fig. 4M). While there appears to exist a possible negative correlation between AAI and geographic distance, we emphasize that this visualization is only exploratory, and rigorous population-genomic analyses — which are beyond the scope of this paper — are needed to quantify any biogeographic structuring.

## Data Records

With the exception of the raw sequencing data, all data files described below are available on a designated Figshare repository^16^, at 10.6084/m9.figshare.30284068. Further, a website providing a hot spring-centric overview of the data is available at: www.loucalab.com/archive/HotSpringsUS. The 500 raw metagenomes are publicly available on the NCBI Sequence Reads Archive (SRA) under BioProjects PRJNA1233593 (years 2020 and 2021)^54^ and PRJNA1233604 (years 2022 and 2023)^55^, run accessions SRR32625235–SRR32625429 and SRR32625235–SRR32625429, respectively. Sample metadata, such as geographic coordinates, physicochemical measurements, hot spring common names and sequencing summary statistics are given in “Data_01 - Sample_metadata.tsv”^16^. Geographic coordinates of samples and basic metadata are also provided in the form of a KML file (“Data_02 - Sample_locations.kml”^16^), which can be loaded into GIS software such as Google Earth. Details about each coassembly, such as N50, L50, maximum contig length and samples used, are given in “Data_03 - Coassembly_metadata.tsv”^16^. The coassembled contigs are available as “Data_04 - Contigs.tar”^16^. Estimated relative abundances of KEGG orthologs in each sample (i.e., functional profiles) are given in “Data_05 - KO_proportions_in_metagenomes.tsv.gz”^16^. Details about each MAG, including contig statistics, tRNA and rRNA genes recovered, taxonomic classification, quality metrics and relative abundance estimates in each sample, are given in “Data_07 - MAG_metadata.tsv”^16^. The 780 MAGs are available as “Data_06 - MAGs.tar”^16^, in the form of a tar archive comprising all MAG fasta files. The MAGs have also been deposited on NCBI Genbank under Bioproject PRJNA1233604, with individual accession numbers listed in “Data_07 - MAG_metadata.tsv”^16^. Relative abundances of MAGs in each sample, based on metagenomic reads recruited, are given as BIOM table “Data_08 - MAG_abundances_per_sample.biom”. Definitions of the SGBs in terms of member MAGs are given in “Data_09 - SGB_members.tsv”^16^. 16S rRNA sequences detected in each MAG are provided as “Data_10 - MAG_16S_sequences.fasta.gz”^16^. A rooted multi-gene phylogenetic tree of bacterial and archaeal MAGs is available as “Data_11 - MAG_tree_rooted_bacteria.tre” and “Data_12 - MAG_tree_rooted_archaea.tre”^16^, respectively. The same trees are also available with taxonomic information, as “Data_13 - MAG_tree_rooted_bacteria_with_taxonomies.tre” and “Data_14 - MAG_tree_rooted_archaea_with_taxonomies.tre”^16^. A table of KEGG orthologs detected in each MAG is given as “Data_15 - KOs_in_MAGs.tsv.gz”. A table listing recruitment rates of quality-filtered reads from each sample to all MAGs is given as “Data_16 - Reads_to_MAGs_recruitment_rates.tsv”. Joint phylogenies of bacterial or archaeal MAGs and GTDB reference genomes are given as “Data_17 - Joint_denovo_bacterial_MAG_GTDB_phylogeny.tre” and “Data_18 - Joint_denovo_archaeal_MAG_GTDB_phylogeny.tre”, respectively.

Throughout our dataset, sample names consist of three period-separated parts, with the first part indicating the hot spring (e.g., AY01), the second part indicating the sampling year (0–4 for 2020–2024) and the third part indicating the specific sample collected from that hot spring in that year (e.g., 01, 02, etc). The number and locations of samples collected in each hot spring differed somewhat each year, hence for example AY01.1.2 and AY01.3.2 originate from the same hot spring (years 2022 and 2023) but not necessarily from the exact same locations. MAG names (e.g., “AA01.MAG06”) always consist of two period-separated parts, with the first part indicating the hot spring from which it was coassembled and the second part being a simple enumeration.

## Data Overview

Our dataset of 500 samples spans temperatures as low as 9.2°C (in shallow pools and outflow streams) up to 93°C (Fig. 1B), pH values from 2.9 up to 9.7 (Fig. 1C), and conductivities ranging from 0.02 to 4.96 mS/cm (Fig. 1D). In particular, 472 out of 500 samples had a measured temperature exceeding 30°C, 421 samples exceeded 40°C and 326 samples exceeded 50°C. The peak source temperature, i.e., the highest source temperature during any survey, exceeded 20°C for all 56 springs, exceeded 30°C for 54 springs, exceeded 40°C for 53 springs and exceeded 50°C for 51 springs.

An overview of our bioinformatics workflow is given in Fig. 2. Our metagenomic dataset comprises a total of 11,485,591,914 raw sequence pairs or about 3.38 Tbp of data (Fig. 3A), with 7,778,111,627 sequence pairs or 1.799 Tbp retained after aggressive quality-filtering (Fig. 3B). Sequences were coassembled into longer contiguous sequences (contigs) separately for each hot spring, i.e., using all sequences from a single hot spring at a time (Fig. 3C). This coassembly strategy was chosen for multiple reasons. First, in contrast to per-sample assembly, coassembly tends to increase sequence contiguity and coverage of MAGs (described below), especially for rare genes or taxa that nevertheless recur across samples^30,56^, and improves MAG binning accuracy thanks to the use of differential coverage metrics^29,57^. Further, co-assembling reads separately from each hot spring avoids cross-contaminating contigs and MAGs with genetic material from geographically separated populations^28^. This safeguard, in turn, will facilitate phylogenomics analyses in individual hot springs and comparative population genomics analyses between hot springs, which are two major applications that we envision for this dataset. Further, generating contigs and MAGs on a per-hot spring basis allows users to cleanly filter data products such as contigs, gene abundance profiles and MAGs based on geographic, geological and environmental criteria depending on research objectives.

Coassembly yielded on average 319,977 contigs at least 500 bp long per hot spring, with an average total contig length of about 486 Mbp per hot spring and an average N50 of 2202 bp (Fig. 3C–J). The longest contigs reached 0.85 Mbp from hot spring AM02 (Soldier Meadows area), 0.73 Mbp from AA03 (Mickey hot springs in Alvord Desert) and 0.69 Mbp from AB03 (near Borax Lake). Protein-coding genes were detected in the contigs and functionally annotated in terms of KEGG orthologs^58^ to generate a gene-centric overview of the functional potential of the microbial communities (Fig. 3K and “Data_05 - KO_proportions_in_metagenomes.tsv.gz”^16^). This revealed that aerobic respiration, fermentation, oxygenic photosynthesis (i.e., *Cyanobacteria*), dissimilatory sulfur and H_2_-based metabolisms as well as anoxygenic phototrophy (e.g., *Chloroflexia*, *Chlorobiia*, Fig. 4K,L) are particularly prevalent in these communities.

Contigs were further binned into MAGs based on tetranucleotide frequencies and differential coverages. Binning yielded 680 bacterial and 100 archaeal MAGs with an estimated completeness ≥ 80% and contamination ≤5%, covering 31 bacterial and 4 archaeal phyla, or 57 bacterial and 8 archaeal classes (overview in Fig. 4 and “Data_07 - MAG_metadata.tsv”^16^). Of these, 127 bacterial and 22 archaeal MAGs are classified as “high quality” following the criteria by^59^, that is, having completeness >90% and contamination <5%, exhibiting 16S, 23S and 5S rRNA genes, and exhibiting elongator tRNA genes for at least 18 amino acids (Fig. 4A–E). Overall, 348 bacterial and 64 archaeal MAGs exhibit at least one copy of the 16S rRNA gene (Fig. 6). In terms of the number of MAGs recovered, the most common bacterial phyla were *Chloroflexota*, *Cyanobacteriota* and *Bacteroidota A*, while nearly all archaeal MAGs belonged to the phylum *Thermoproteota* (Fig. 4I). These phyla also dominate in terms of their relative abundances based on fractions of metagenomic reads recruited to MAGs (Fig. 5). On average 13% of quality-filtered metagenomic reads per sample could be confidently mapped back to these MAGs, indicating that the diversity in many of these hot springs is considerably higher than represented by the MAGs. Genome sizes estimated from MAG sizes and MAG completeness varied widely, with members of the phylum *Patescibacteria* generally having the smallest genomes, often below 1 Mbp (Fig. 4I). GC content also varied greatly between MAGs, ranging from just under 0.3 to over 0.7 (Fig. 4J). The temperature of the sample in which each MAG was found at highest relative abundance, which may be seen as a rough proxy of an organism’s ideal growth temperature, also varied considerably (Fig. 4N), with the median being 55.4°C. To assess the species-level diversity among our MAGs, we also clustered MAGs into species-level genome bins (SGBs) based on an average nucleotide identity of 95%^45,46^, yielding 347 bacterial and 58 archaeal SGBs. Of these, 275 SGBs comprised only a single MAG, while 6 of the SGBs comprised 10 or more MAGs (Fig. 4F and “Data_09 - SGB_members.tsv”^16^). In all cases, MAGs associated with an SGB originated from distinct hot springs, thus enabling biogeographic analyses of the intraspecific diversity in each SGB.

The recovered MAGs increase our public repertoire and taxonomic breadth of genome-sequenced prokaryotes from extreme environments. Indeed, 531 of the MAGs could only be placed into a known genus (but not species), 82 MAGs could only be placed into a known family (but not genus), and 4 MAGs could only be placed into a known order (but not family), based on a joint phylogeny with the comprehensive Genome Taxonomy Database (GTDB) r226 and even when considering candidatus and other informal/putative taxa^60^ (Fig. 6 see “Data_17 - Joint_denovo_bacterial_MAG_GTDB_phylogeny.tre”^16^ and “Data_18 - Joint_denovo_archaeal_MAG_GTDB_phylogeny.tre”^16^ for phylogenetic placements). When only considering placements in properly named taxa (i.e., with cultured representatives), 286 MAGs could only be placed into a proper genus, 258 MAGs into a proper family, 96 MAGs into a proper order, 83 MAGs into a proper class, 23 into a proper phylum and 1 MAG merely into the domain bacteria. The latter, AB02.MAG08, was recovered from a small hot spring near Borax Lake, Oregon, with a source temperature last recorded at 78.9°C (Fig. 1F). These results suggest that AB02.MAG08 originates from a phylum with no cultured representative in the GTDB, and that an additional 23 MAGs originate from classes with no cultured representatives in the GTDB. When we mapped these MAGs to all properly named GTDB reference genomes based on average amino-acid identity (AAI), the AAIs to the closest hits ranged from 38.6% to 60.2% (Fig. 4H), which are comparable to empirical AAI thresholds for delineating prokaryotic phyla or classes^61^. Combined with our provided functional annotations, such MAGs can yield insight into the possible metabolisms of poorly explored taxa. For example, AB02.MAG08 exhibited several genes known to be involved in nitrate respiration, nitrite respiration and reductive acetogenesis, indicating an anaerobic lifestyle. The presented metagenomes and MAGs also expand our ability to study the microbial ecology and evolution in these island-like environments, for example via phylogeographic analyses^3^ and population genomics^62^, and could guide future culturing efforts based on inferred nutrient requirements^63^. As several of the hot springs (corresponding to a total of 573 MAGs) were surveyed on all 4 years, this also presents an opportunity to examine genome sequence evolution through time, for example via phylogenomics^64^.

## Technical Validation

The completeness and contamination level of each MAG was assessed using CheckM2^37^. The number of tRNA genes in each MAG was determined using trnascan-se^44^. rRNA genes were detected in each MAG using barrnap. Coverages of contigs in the full coassemblies and in MAGs specifically were determined using Bowtie2^26^ and samtools^27^. Standard summary statistics such as contig L50, N50 and total contig length were computed from the fasta files using python. Command line arguments and version numbers of software used are detailed in the Methods.

## Data Availability

Novel sequencing data generated have been made publicly available on designated repositories, described in detail in our Data Records section. Briefly, the raw sequencing data are publicly available on the NCBI Sequence Reads Archive (SRA) under BioProjects PRJNA1233593 (years 2020 and 2021) and PRJNA1233604 (years 2022 and 2023), the 780 MAGs are publicly available on Genbank under BioProject PRJNA1233604 (https://identifiers.org/ncbi/bioproject:PRJNA1233604), and all other data are available on Figshare (10.6084/m9.figshare.30284068).

## Code availability

All software used in this paper, including versions and non-default command-line arguments, have been described in the Methods and are freely available online.
